# Geographic Disparities in Previously Diagnosed Health Conditions in Colorectal Cancer Patients Are Largely Explained by Age and Area Level Disadvantage

**DOI:** 10.3389/fonc.2018.00372

**Published:** 2018-09-11

**Authors:** Belinda C. Goodwin, Sonja March, Michael J. Ireland, Fiona Crawford-Williams, Shu-Kay Ng, Peter D. Baade, Suzanne K. Chambers, Joanne F. Aitken, Jeff Dunn

**Affiliations:** ^1^Institute for Resilient Regions, University of Southern Queensland, Springfield Central, Toowoomba, QLD, Australia; ^2^School of Psychology and Counseling, University of Southern Queensland, Springfield Central, Toowoomba, QLD, Australia; ^3^Menzies Health Institute, Griffith University, Southport, QLD, Australia; ^4^Cancer Research Centre, Cancer Council Queensland, Fortitude Valley, QLD, Australia; ^5^Prostate Cancer Foundation of Australia, St Leonards, NSW, Australia; ^6^Exercise Medicine Research Institute, Edith Cowan University, Perth, WA, Australia; ^7^School of Public Health Fand Social Work, Queensland University of Technology, Brisbane, QLD, Australia; ^8^Menzies Health Institute Queensland, Griffith University, Brisbane, QLD, Australia; ^9^School of Medicine, Griffith University, Brisbane, QLD, Australia

**Keywords:** colorectal cancer, comorbidity, regional disparity, socio-economic status, rural health

## Abstract

**Background:** Geographical disparity in colorectal cancer (CRC) survival rates may be partly due to aging populations and disadvantage in more remote locations; factors that also impact the incidence and outcomes of other chronic health conditions. The current study investigates whether geographic disparity exists amongst previously diagnosed health conditions in CRC patients above and beyond age and area-level disadvantage and whether this disparity is linked to geographic disparity in CRC survival.

**Methods:** Data regarding previously diagnosed health conditions were collected via computer-assisted telephone interviews with a cross-sectional sample of *n* = 1,966 Australian CRC patients between 2003 and 2004. Ten-year survival outcomes were acquired in December 2014 from cancer registry data. Multivariate logistic regressions were applied to test associations between previously diagnosed health conditions and survival rates in rural, regional, and metropolitan areas.

**Results:** Results suggest that only few geographical disparities exist in previously diagnosed health conditions for CRC patients and these were largely explained by socio-economic status and age. Living in an inner regional area was associated with cardio-vascular conditions, one or more respiratory diseases, and multiple respiratory diagnoses. Higher occurrences of these conditions did not explain lower CRC-specific 10 years survival rates in inner regional Australia.

**Conclusion:** It is unlikely that health disparities in terms of previously diagnosed conditions account for poorer CRC survival in regional and remote areas. Interventions to improve the health of regional CRC patients may need to target issues unique to socio-economic disadvantage and older age.

## Introduction

There is growing concern over health disparities between rural, regional, and metropolitan communities in Australia and internationally (1–4). Evidence suggests that individuals living in geographically remote areas experience higher incidence of morbidity and mortality than their metropolitan counterparts (1, 5–7). Living in a non-metropolitan area has been associated with higher incidences of a number of serious health conditions including cancer (2, 6–8), diabetes, and cardiovascular disease (9, 10). In terms of mortality, geographical remoteness is also associated with lower chance of survival for those diagnosed with many serious health conditions (11, 12). In particular, it has been estimated that patients with colorectal cancer (CRC) residing in non-metropolitan areas have between a 4 and 30% higher chance of dying from the disease, within 5 years of diagnosis, than metropolitan residents (13, 14).

Geographical health disparities are thought to be due, not only to limited physical access to health care, but also to differences in demography, attitudes, lifestyle factors, and cultural practices in regional and rural settings (5, 6, 15). However, it is unclear whether these factors provide a full explanation for regional disparity in CRC outcomes. Individuals living in regional and remote areas exhibit poorer health profiles (e.g., more likely to experience multiple chronic conditions) than those living in metropolitan areas (2, 7–10). Pre-existing comorbid health conditions often lead to later CRC detection, make patients less suitable candidates for curative surgery, and increase the chance of mortality by approximately 20% (16–18). Given these risks, it is plausible that geographical disparity in CRC outcomes may be somewhat accounted for by a tendency for regional and remote patients to report multiple health problems.

Remote and regional areas also tend to have different demographic profiles than major cities, with higher proportions of older residents and higher area level disadvantage (19); factors that contribute to CRC incidence and mortality as well as comorbidity and poorer health in general (16, 20, 21). Average age and socio-economic status (SES) in regional and remote areas are likely to account for geographical disparity in the diagnosis of other health conditions among CRC patients. In order to identify and address the causes of geographical disparities in the health and mortality of CRC patients, it is therefore important to establish whether these disparities exist above and beyond age and area level disadvantage. Without this knowledge, those working to improve outcomes for non-metropolitan CRC patients, may concentrate efforts toward increasing healthcare access in remote areas and fail to address the health issues associated with socio-economic disadvantage and older age.

Understanding the risk factors associated with other diagnosed health conditions among CRC patients is an important focus for health researchers and policymakers who wish to improve cancer outcomes. To date, no study has measured comorbid (or premorbid) conditions in rural, regional, and metropolitan CRC patients, nor has their impact on geographical disparities in CRC survival rates been empirically tested. The aim of this study is to determine whether geographical disparities in previously diagnosed chronic health conditions exist above and beyond the age and SES of CRC patients and whether such disparities play a role in reduced CRC survival rates in regional and remote areas compared to major cities.

## Methods

### Participants and procedure

Data collection for this study formed part of the Colorectal Cancer and Quality of Life Study (for recruitment details see (22)). Approval for the use of these data was granted by the University's Human Research Ethics Committee (Approval Number: H17-REA014). Participants had a primary diagnosis of CRC between 1 January 2003 and 31 December 2004, were between the ages of 20 and 80 years, 59.8% male and reasonably evenly distributed across SES deciles. The most common cancer site was the colon (70%). Table 1 provides a breakdown of participant demographics, cancer site, and stage information for the current sample.

**Table 1:** Sample characteristics (*n* = 1,966).

	***n* (%)**
**SEX**	
Female	790 (40%)
**AGE**	
20–49 yrs	167 (8.5%)
50–59 yrs	382 (19.4%)
60–69 yrs	665 (33.8%)
70–79 yrs	752 (38.3%)
**SES QUINTILE**	
(lowest) 1	334 (17.0%)
2	325 (16.5%)
3	365 (18.6%)
4	651 (33.1%)
(highest) 5	288 (14.6%)
**LOCATION (ASGC-RA)**	
Metropolitan	981 (49.9%)
Inner regional	622 (31.6%)
Outer regional	336 (17.1%)
Remote	19 (1.0%)
Very Remote	8 (0.4%)
**SITE**	
Colon	1203 (69.9%)
Rectum	519 (30.1%)
**STAGE (DUKES)**	
A	437 (29.4%)
B	521 (35.1%)
C	487 (32.8%)
D	40 (2.7%)

Participants were invited (*n* = 3,182) to the research via mail through their treating doctor who consented to and endorsed the invitation. Participants who responded and provided informed consent (*n* = 2181) were then contacted by phone to complete a computer assisted telephone interview (CATI), 90% (*n* = 1,966) of the respondents completed the interview comprising the sample for the current study.

Representativeness of the sample was evaluated by comparing gender, age, and disease specific information from the current sample to those who were eligible, but were not recruited to the study. There were no gender differences, however, 70–80 years old CRC survivors were under represented along with those with rectal and advanced stage cancer (*p* = < 0.05).

### Measures

#### Demographics

Each respondent provided their gender, date of birth, and postal address. Participant location of residence was categorized as major city; inner regional; outer regional; remote; or very remote based on the Australian Standard Geographical Classification - Remoteness Area (ASGC-RA; AGDHA 2006). For the purposes of this study, major city residents were coded as (1) major city (*N* = 981); (2) inner regional coded as regional (*N* = 622) and the remainder coded as (3) outer regional and remote (*N* = 363) as per methods used in Australian Institute of Health and Welfare (AIHW) studies in 2008 (23). Each participant was assigned a percentile rank based on the Socio-Economic Index for Areas (SEIFA); which reflected their estimated socio-economic status (SES) according to postcode; a rank of 1 indicating that the participant resided in an area assigned the lowest SES and a rank of 100 reflecting the highest.

#### Previously diagnosed conditions

Participants were asked to identify (on a checklist—see Table 2) any health conditions they had previously been diagnosed with^1^. Body mass index (BMI) for each participant was calculated using their self-reported weight (prior to diagnosis) and height. According to World Health Organization recommendations, participants with a BMI over 30 were classified as obese.

**Table 2:** Descriptive statistics of sample and Zero-order correlations between premorbid conditions, SES and age.

	**Descriptive statistics**		**Bivariate correlations (r)**	
	**Mean (*SD*)**	**%**	**SES**	**Age**
SES	54.00 (26.26)		1	
age	65.01 (10.35)		−0.039	1
Any cardiovascular		59.4	−0.033	0.284^**^
Heart attack		7.7	−0.070^**^	0.158^**^
Angina Pectoris		9.9	−0.084^**^	0.174^**^
High blood pressure		40.9	−0.060^**^	0.222^**^
High cholesterol		28.7	0.003	0.142^**^
Other heart condition		15.6	−0.013	0.183^**^
Stroke		4.1	0.011	0.115^**^
Any respiratory		17.9	0.007	0.061^**^
Asthma		12.7	0.021	0.024
Chronic Bronchitis		6.2	0.003	0.035
Emphysema		3.6	−0.022	0.094^**^
Any skeletal		25.2	−0.009	0.218^**^
Osteoporosis		5.9	0.021	0.145^**^
Osteoarthritis		16.5	−0.020	0.163^**^
Rheumatoid Arthritis		5.4	−0.019	0.065^**^
Leukemia		0.8	−0.022	0.035
Any other		62.4	−0.045^*^	0.020
Diabetes		12.6	−0.027	0.118^**^
Ulcer		12.4	0.014	0.076^**^
Migraine		12.8	0.004	−0.024
Depression		13.5	−0.010	−0.073^**^
Any previous cancer		20.0	−0.066^**^	0.058^*^
Obese		25.5	−0.019	−0.109^**^
Other serious illness		1.9	−0.002	0.054^*^
Any condition		87.6	−0.055^*^	0.224^**^

** *< p = 0.01*,

* *< p = 0.05*.

#### Stage and 10-years censored mortality data (all cause and CRC specific)

The mortality status of each participant at the 30th of December 2013 was obtained from the relevant State Government Department of Births, Deaths, and Marriages along with stage of cancer at diagnosis. This information includes date and cause of death and is checked against data from hospitals, nursing homes, coroners, the National Death Index, and the Australian Bureau of Statistics. A censored mortality variable was created that indicated whether or not the patient had passed away by the end of the study. Elapsed time since diagnosis was defined as total days from patient diagnosis to death or to the end of the study—whichever came first.

### Clustering of conditions

We employed two methods of grouping health conditions for analytic purposes (the first intuitive and the second statistical). Firstly, we categorized conditions according to the physical site or system in the body which that condition effects (i.e., cardiovascular, respiratory, skeletal, and other conditions) and coded participants according to whether they reported one or more of each type of condition (see Table 2). Secondly, we investigated multimorbidity through a clustering method of pairwise concordance statistics (24). This method adopts the asymmetric Somers' D statistic to quantify the degree of “non-random” multimorbidity, also known as “associative multimorbidity” (25). Identification of significant (non-random) multimorbidity between conditions is an informative way to view disease patterns and indicates a potential sharing of risk factors of the diseases (26, 27). The clustering method adopts the Benjamini-Hochberg procedure to control for the false discovery rate, offering protection against false positives (28). The use of UCINET6 for Windows (29) can graphically display the identified multimorbidity patterns of health conditions, where any two conditions form the “closest” pair when their pairwise Somers' D statistic is maximum and significant. Presence or absence of comorbid conditions for each participant were saved as variables for analysis. These two grouping methods allowed us to draw conclusions regarding the likelihood of rural, regional, and metropolitan CRC patients reporting a certain type of condition, as well as the likelihood of reporting multiple commonly co-occurring conditions.

### Data analysis

Contrast variables (see Table 2 notes) were created in order to apply all three comparisons between major city, regional, and rural groups in separate analyses. Logistic regression was applied to estimate the odds ratio (OR) associated with each condition, each type of condition, and multimorbidity cluster membership across rural, regional, and major cities. Unadjusted ORs and 95% confidence intervals were reported alongside those adjusted for age and SES. Cox regressions were then conducted to estimate hazard ratios (HR) and 95% confidence intervals associated with patient location of residence, controlling for stage of cancer at diagnosis, age, and SES. Mediations were tested through a series of Cox regression models and Sobel tests to assess significance of the indirect effect. All main analyses were performed in IBM SPSS version 23 (30).

## Results

Table 2 reports the percentage of participants that reported a previous diagnosis of each condition. The most commonly reported condition was high blood pressure (40.9%), followed by high cholesterol (28.7%), obesity (25.5%), and a previous cancer diagnosis (20.0%). One or more cardiovascular-related diagnoses were reported by 59.4% of participants, while 17.9% reported at least one respiratory condition and 25.2% reported at least one skeletal condition. Eighty-seven percent of the sample reported being previously diagnosed with at least one condition overall.

Preliminary analyses of variance showed that remoteness of living was negatively associated with SES and the patients living in regional areas were significantly older than those in metropolitan or rural areas. As shown in Table 2, SES was weakly, but significantly negatively associated with a previous diagnosis of heart attack (*r* = −0.07, *p* < 0.01), angina pectoris (*r* = −0.08, *p* < 0.01), high blood pressure (*r* = −0.06, *p* < 0.01), one or more other serious illness (*r* = −0.05, *p* < .05), previous cancer diagnosis (*r* = −0.07, *p* < 0.01), and one more previously diagnosed condition overall (*r* = −0.06, *p* < 0.05). Based on these results, which suggest that SEIFA rank and age are related to both remoteness and several premorbid diagnoses, crude ORs as well as age- and SES-adjusted ORs are presented in the main analysis.

### Main analyses

#### Regional vs. major city

As shown in Table 3, crude logistic regression results suggest that when compared to CRC patients living in major cities, regional CRC patients were significantly more likely to report having had a heart attack (OR = 1.81, 95% CI = 1.13–2.92), other heart condition (OR = 1.53, 95% CI = 1.07–2.18), one or more respiratory conditions (OR = 1.45, 95% CI = 1.03–2.06), or emphysema (OR = 2.37, 95% CI = 1.09–5.12). When adjusted for age, ORs became insignificant for other heart conditions (OR = 1.36, 95% CI = 0.95–1.96) and marginally significant for heart attack (OR = 1.59, 95% CI = 0.98–2.59), one or more respiratory conditions (OR = 1.40, 95% CI = 0.99–1.98) and emphysema (OR = 2.11, 95% CI = 0.97–4.58). These marginally significant results remained when ORs were adjusted for both age and SEIFA rank. In the case of emphysema, the age and SES adjusted OR was not significant. A suppression effect (31) of SEIFA rank was apparent in the case of stroke (OR = 0.46, 95% CI = 0.21–0.98), whereby adjusting the OR for SES *increased* significance. That is, although previous stroke diagnosis is not associated with the variance in location (regional vs. major city) that is shared with SES, it is associated with the variance in location that is not shared with SES.

**Table 3:** Odds Ratios (and 95% Confidence Intervals) for reporting each of the listed pre-morbid health conditions for rural, regional, and metropolitan CRC patients (unadjusted and controlling for age and SES).

	**OR (CI, 95%)**								
	**Regional vs. Metro^a^**			**Rural vs. Metro^b^**			**Rural vs. Regional^c^**		
	**Unadjusted**	**Adjusted^age^**	**Adjusted^age, SES^**	**Unadjusted**	**Adjusted^age^**	**Adjusted^age, SES^**	**Unadjusted**	**Adjusted^age^**	**Adjusted^age, SES^**
Any cardiovascular	1.09 (0.83, 1.44)	0.93 (0.70, 1.24)	0.89 (0.66, 1.20)	0.96 (0.72, 1.32)	1.11 (0.80, 1.55)	1.09 (0.78, 1.51)	0.69 (0.41, 1.17)	0.83 (0.49, 1.44)	0.99 (0.53, 1.60)
Heart attack	**1.81 (1.13, 2.92)**	1.59 (0.98, 2.59)^∧^	1.34 (0.81, 2.23)	0.75 (0.41, 1.37)	0.89 (0.48, 1.65)	0.81, (0.43, 1.50)	0.36 (0.11, 1.06)	0.47 (0.15, 1.47)	0.65 (0.22, 1.91)
Angina pectoris	1.35 (0.86, 2.11)	1.18 (0.75, 1.86)	0.89 (0.55, 1.44)	0.61 (0.34, 1.07)	0.71 (0.40, 1.26)	0.60 (0.34, 1.08)	0.77 (0.29, 2.07)	1.15 (0.65, 2.04)	1.14 (0.46, 2.86)
High blood pressure	1.00 (0.76, 1.30)	0.88 (0.67, 1.17)	0.81 (0.60, 1.08)	1.16 (0.85, 1.58)	1.32 (0.96, 1.82)	1.25 (0.90, 1.72)	1.04 (0.62, 1.76)	1.24 (0.73, 2.12)	1.38 (0.80, 2.39)
High cholesterol	0.82 (0.61, 1.10)	0.75 (0.56, 1.02)^∧^	0.75 (0.55, 1.03)	1.19 (0.85, 1.66)	1.30 (0.92, 1.82)	1.30 (0.92, 1.83)	1.10 (0.62, 1.94)	1.24 (0.70, 2.18)	1.21 (0.68, 2.15)
Other heart condition	**1.53 (1.07, 2.18)**	1.36 (0.95, 1.96)	1.40 (0.96, 2.05)	0.78 (0.50, 2.18)	0.89 (0.57, 1.39)	0.91 (0.58, 1.42)	**0.35 (0.15, 0.83)**	0.44 (0.19, 1.01)^∧^	0.42 (0.18, 1.00) ^∧^
Stroke	0.53 (0.25, 1.10)	**0.46 (0.22, 0.95)**	**0.46 (0.21, 0.98)**	1.40 (0.66, 2.96)	1.68 (0.79, 3.59)	1.69 (0.78, 3.63)	1.33 (0.38, 4.64)	1.63 (0.48, 5.50)	1.55 (0.44, 5.42)
Any respiratory	**1.45 (1.03, 2.06)**	1.40 (0.99, 1.98)^∧^	1.42 (0.99, 2.05)^∧^	**0.60 (0.39, 0.92)**	**0.62 (0.40, 0.96)**	**0.63 (0.40, 0.97)**	**0.32 (0.14, 0.76)**	**0.35 (0.15, 0.82)**	**0.33 (0.14, 0.81)**
Asthma	1.24 (0.83, 1.84)	1.22 (0.82, 1.82)	1.31 (0.86, 1.98)	0.77 (0.47, 1.24)	0.78 (0.48, 1.26)	0.82 (0.50, 1.33)	0.47 (0.19, 1.18)	0.49 (0.19, 1.22)	0.42 (0.16, 1.12)
Chronic Bronchitis	1.68(0.97, 2.89)^∧^	1.62 (0.94, 2.80)	1.71 (0.96, 3.03)^∧^	0.62 (0.31, 1.26)	0.65 (0.32, 1.31)	0.67 (0.33, 1.36)	**0.20 (0.05, 0.86)**	**0.22 (0.50, 0.92)**	**0.19 (0.40, 0.87)**
Emphysema	**2.37 (1.09, 5.12)**	2.11 (0.97, 4.58)^∧^	1.91 (0.85, 4.25)	**0.29 (0.09, 0.90)**	0.34 (0.11, 1.04)^∧^	**0.32 (0.10, 1.00)**	**0.08 (0.01, 0.77)**	0.11 (0.01, 1.04)^∧^	0.15 (0.02, 1.41)
Any skeletal	1.16 (0.85, 1.56)	1.02 (0.74, 1.39)	1.02 (0.74, 1.42)	0.96 (0.67, 1.36)	1.11 (0.77, 1.60)	1.11 (0.77, 1.61)	0.76 (0.41, 1.40)	0.91 (0.49, 1.70)	0.90 (0.48, 1.69)
Osteoporosis	0.65 (0.35, 1.20)	0.55 (0.30, 1.03)^∧^	0.56 (0.30, 1.07)	0.97 (0.49, 1.90)	1.17 (0.59, 2.32)	1.18 (0.59, 2.36)	1.12 (0.35, 3.53)	1.41, (0.46, 4.33)	1.33 (0.42, 4.23)
Osteoarthritis	1.28 (0.90, 1.83)	1.15 (0.80, 1.66)	1.10 (0.75, 1.60)	0.73 (0.48, 1.13)	0.82 (0.53, 1.28)	0.80 (0.51, 1.25)	0.49 (0.22, 1.11)	0.59 (0.27, 1.32)	0.63 (0.28, 1.42)
Rheumatoid arthritis	1.39 (0.81, 2.39)	1.39 (0.81, 2.39)	1.48 (0.83, 2.61)	1.60 (0.86, 2.97)	1.60 (0.86, 2.97)	1.66 (0.88, 3.11)	0.78 (0.26, 2.32)	0.87 (0.30, 2.52)	0.83 (0.28, 2.51)
Leukemia	0.67 (0.14, 3.25)	0.61 (0.13, 2.97)	0.47 (0.09, 2.36)	1.09 (0.20, 6.03)	1.22 (0.22, 6.79)	1.05 (0.19, 5.89)	4.61 (0.64, 33.17)	5.02 (0.71, 35.40)	5.61 (0.86, 36.21)^∧^
Any other	1.00 (0.76, 1.32)	0.99 (0.76, 1.31)	0.91 (0.68, 1.21)	1.03 (0.75, 1.41)	1.04 (0.76, 1.43)	0.98 (0.71, 1.36)	0.86 (0.51, 1.46)	0.87 (0.51, 1.49)	0.96 (0.56, 1.66)
Diabetes	0.80 (0.53, 1.20)	0.73 (0.48, 1.10)	0.66 (0.43, 1.02)^∧^	1.18 (0.75, 1.85)	1.30 (0.83, 2.06)	1.23 (0.78, 1.96)	1.24 (0.59, 2.63)	1.41 (0.67, 2.96)	1.52 (0.73, 3.20)
Stomach ulcer	1.14 (0.77, 1.68)	1.08 (0.73, 1.59)	1.21 (0.80, 1.82)	1.23 (0.79, 1.91)	1.31 (0.84, 2.04)	1.40 (0.89, 2.21)	0.60 (0.26, 1.38)	0.66 (0.29, 1.50)	0.55(0.23, 1.33)
Migraine headaches	1.23 (084, 1.82)	0.95 (0.60, 1.50)	1.32 (0.88, 1.98)	0.96 (0.61, 1.52)	0.95 (0.60, 1.50)	0.99 (0.98, 1.01)	0.67 (0.29, 1.53)	0.65 (0.28, 1.49)	0.61 (0.26, 1.45)
Depression	1.09 (0.74, 1.63)	1.16 (0.78, 1.72)	1.07 (0.71, 1.63)	0.69 (0.43, 1.12)	0.66 (0.41, 1.07)	0.63 (0.39, 1.02)	1.03 (0.47, 2.27)	0.95 (0.43, 2.13)	1.06 (0.48, 2.35)
Any previous cancer	1.02 (0.73, 1.42)	0.98 (0.71, 1.37)	0.84 (0.60, 1.19)	1.00 (0.68, 1.46)	1.03 (0.70, 1.51)	0.94 (0.64, 1.38)	1.03 (0.54, 1.96)	1.09 (0.57, 2.06)	1.30 (0.69, 2.44)
Obese	0.75 (0.55, 1.02)^∧^	0.79 (0.58, 1.08)	0.76 (0.55, 1.05)	1.34 (0.95, 1.89)	1.28 (0.91, 1.81)	1.25 (0.88, 1.77)	1.36 (0.79, 2.45)	1.28 (0.72, 2.27)	1.34 (0.75, 2.40)
Other serious illness	0.99 (0.39, 2.57)	0.90 (0.35, 2.34)	0.93 (0.34, 2.53)	1.28 (0.44, 3.68)	1.43 (0.49, 4.15)	1.46 (0.50, 4.31)	1.39 (0.26, 7.33)	1.57 (0.31, 7.99)	1.53 (0.29, 8.06)
Any condition	1.06 (0.70, 1.59)	0.88 (0.58, 1.35)	0.77 (0.50, 1.20)	1.06 (0.66, 1.70)	1.22 (0.75, 1.98)	1.11 (0.68, 1.20)	0.61 (0.29, 1.25)	0.74 (0.34, 1.61)	0.84 (0.37, 1.89)

Contrast variable coding:

a Metro = −0.5, Regional = 0.5, Rural = 0.

b Metro = −0.5, Regional = 0, Rural = 0.5.

c *Metro = 0, Regional = −0.5, Rural = 0.5; bold = significant at p < 0.05; ^∧^ = marginally significant, p < 0.075*.

#### Rural vs. major city

Rural CRC patients were significantly less likely than major city dwellers to report a previous diagnosis of one or more respiratory conditions (OR = 0.60, 95% CI = 0.39–0.92) or emphysema (OR = 0.29, 95% CI = 0.09–0.90). The age and SES adjusted ORs for respiratory conditions (OR = 0.63, 95% CI = 0.40–0.97) and emphysema (OR = 0.32, 95% CI = 0.10–1.00) remained significant suggesting that these factors do not account for any of the effects of location on diagnosis. No other significant differences in diagnoses were found between rural and major city patients.

#### Rural vs. regional

Rural patients were significantly less likely to report a previous diagnosis of other heart conditions (OR = 0.35, 95% CI = 0.15–0.83), any respiratory disease (OR = 0.32, 95% CI = 0.14–0.76), chronic bronchitis (OR = 0.20, 95% CI = 0.05–0.86) and emphysema (OR = 0.08, 95% CI = 0.01–0.77) when compared to regional patients. These effects remained significant, or marginally significant, when ORs were adjusted for age and SES.

### Multimorbidity analysis

**Figure 1:**
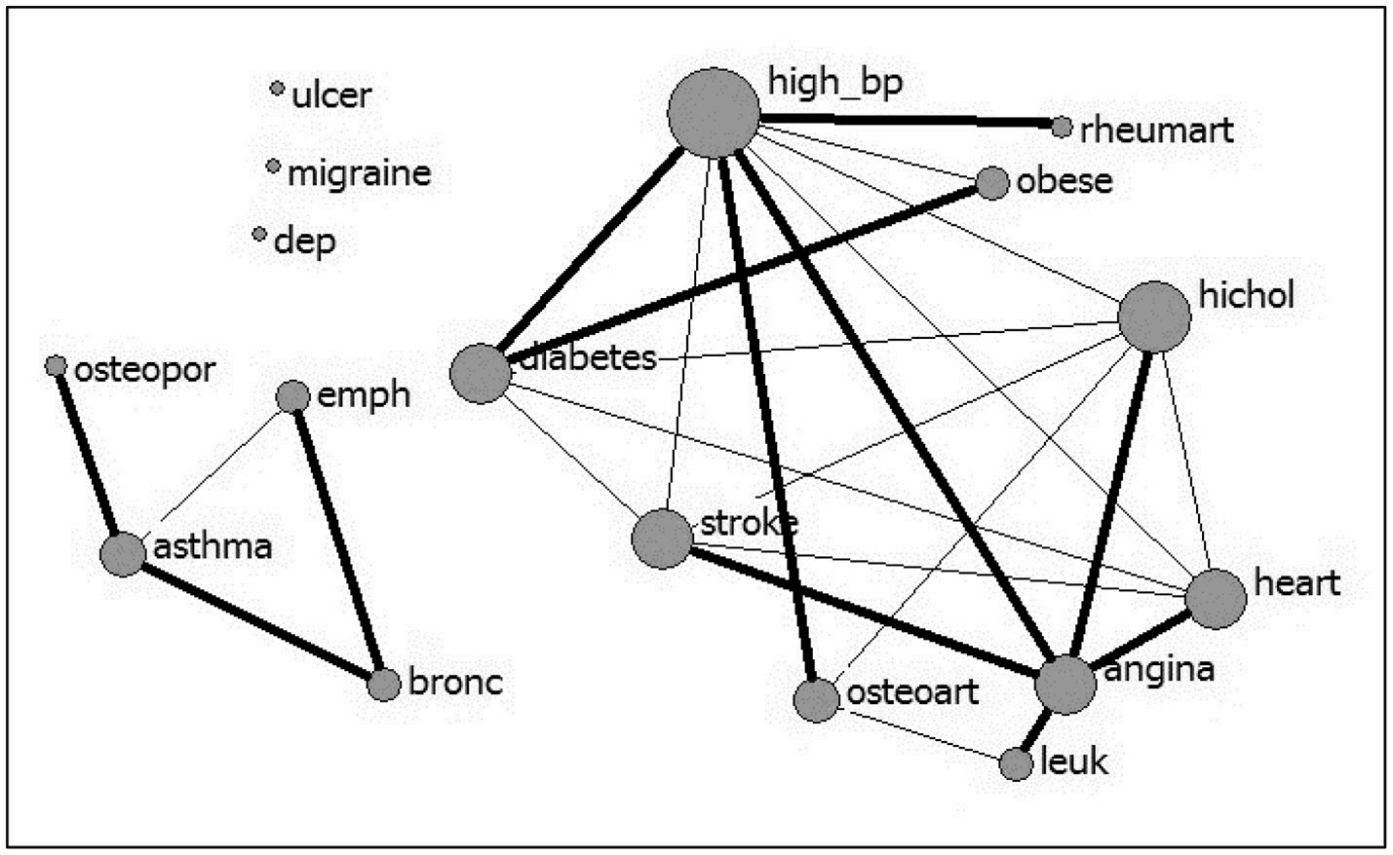
Multimorbidity between 17 conditions (nodal size is proportional to the number of conditions comorbid with the condition; bolded lines link the “closest” pairs).

Multimorbidity analysis identified five overlapping clusters of conditions (see Table 4). High blood pressure and high cholesterol showed the highest number of comorbid conditions, followed by heart attack, angina pectoris, stroke, and diabetes (see Figure 1). Most of the overlapping clusters related to conditions of the same type (e.g., cardiovascular disease group: heart attack, angina pectoris, high blood pressure, high cholesterol, and stroke), however, there were links with diabetes as well as osteoarthritis with the cardiovascular disease group. Further, a respiratory cluster (asthma, chronic bronchitis, emphysema) and a cluster associating high blood pressure, diabetes, and obesity were identified.

**Table 4:** Five overlapping clusters of conditions.

**Cluster label**	**Conditions on cluster**	**Strength of multimorbidity**
**Cluster 1** (CVD)	Heart attack, Angina pectoris, High blood pressure, High cholesterol, Stroke	0.250
**Cluster 2** (CVD + diabetes)	Heart attack, High blood pressure, High cholesterol, Stroke, Diabetes	0.199
**Cluster 3** (Respiratory)	Asthma, Chronic bronchitis, Emphysema	0.249
**Cluster 4** (HBP + diabetes + obese)	High blood pressure, Diabetes, Obese	0.200
**Cluster 5** (CVD + arthritis)	High blood pressure, High cholesterol, Osteoarthritis	0.175

As shown in Table 5, only membership in the respiratory cluster significantly differed according to location. That is, adjusting for age and SES, regional CRC patients were more likely to report multiple respiratory diagnoses compared to metropolitan patients (HR = 1.92, 95% CI = 1.05–1.3.51). Also, rural patients were marginally less likely than regional patients to report diagnosis of multiple respiratory disorders (HR = 0.36, 95% CI = 0.12–1.03).

**Table 5:** Odds Ratios (and 95% Confidence Intervals) for multimorbidity cluster membership in rural, regional, and metropolitan CRC patients (unadjusted and controlling for age and SES).

	**OR (CI, 95%)**								
	**Regional vs. Metro**^**a**^			**Rural vs. Metro**^**b**^			**Rural vs. Regional**^**c**^		
	**Unadjusted**	**Adjusted^age^**	**Adjusted^age, SES^**	**Unadjusted**	**Adjusted^age^**	**Adjusted^age, SES^**	**Unadjusted**	**Adjusted^age^**	**Adjusted^age, SES^**
Cluster 1 (CVD)	1.02 (0.81, 1.29)	0.97 (0.76, 1.23)	0.83 (0.63, 1.9)	1.05 (0.80, 1.37)	1.10 (0.83, 1.45)	0.97 (0.71, 1.32)	0.93 (0.63, 1.38)	0.96 (0.64, 1.44)	1.18 (0.76, 1.84)
Cluster 2 (CVD + diabetes)	0.41 (0.61, 1.22)	0.83 (0.59, 1.17)	0.73 (0.50, 1.08)	0.98 (0.66, 1.45)	1.02 (0.68, 1.52)	0.94 (0.61, 1.46)	1.20 (0.68, 2.11)	1.23 (0.70, 2.18)	1.44 (0.77, 2.67)
Cluster 3 (Respiratory)	1.61^∧^ (0.95, 2.75)	1.59 (0.94, 2.71)	1.92 (1.05, 3.51)	1.11 (0.60, 2.05)	1.12 (0.60, 2.07)	1.20 (0.61, 2.36)	0.47 (0.18, 1.19)	0.47 (0.19, 1.21)	0.36^∧^ (0.12, 1.03)
Cluster 4 (HBP+ diabetes+ obese)	1.04 (0.81, 1.33)	1.02 (0.79, 1.31)	0.87 (0.66, 1.16)	1.15 (0.87, 1.54)	1.17 (0.88, 1.03)	1.03 (0.76, 1.42)	0.94 (0.62, 1.43)	0.95 (0.63, 1.46)	1.18 (0.75, 1.87)
Cluster 5 (CVD+arthritis)	1.10 (0.80, 1.51)	1.06 (0.77, 1.45)	0.91 (0.64, 1.29)	0.92 (0.64, 1.33)	0.96 (0.66, 1.40)	0.81 (0.53, 1.22)	0.83 (0.49, 1.41)	0.85 (0.49, 1.45)	1.05 (0.59, 1.88)
Any multimorbidity	1.17 (0.96, 1.44)	1.12 (0.90, 1.38)	1.10 (0.87, 1.39)	1.06 (0.83, 1.33)	1.08 (0.85, 1.38)	1.05 (0.81, 1.37)	0.94 (0.62, 1.43)	0.95 (0.63, 1.46)	1.18 (0.75, 1.87)

### Survival analysis

**Figure 2:**
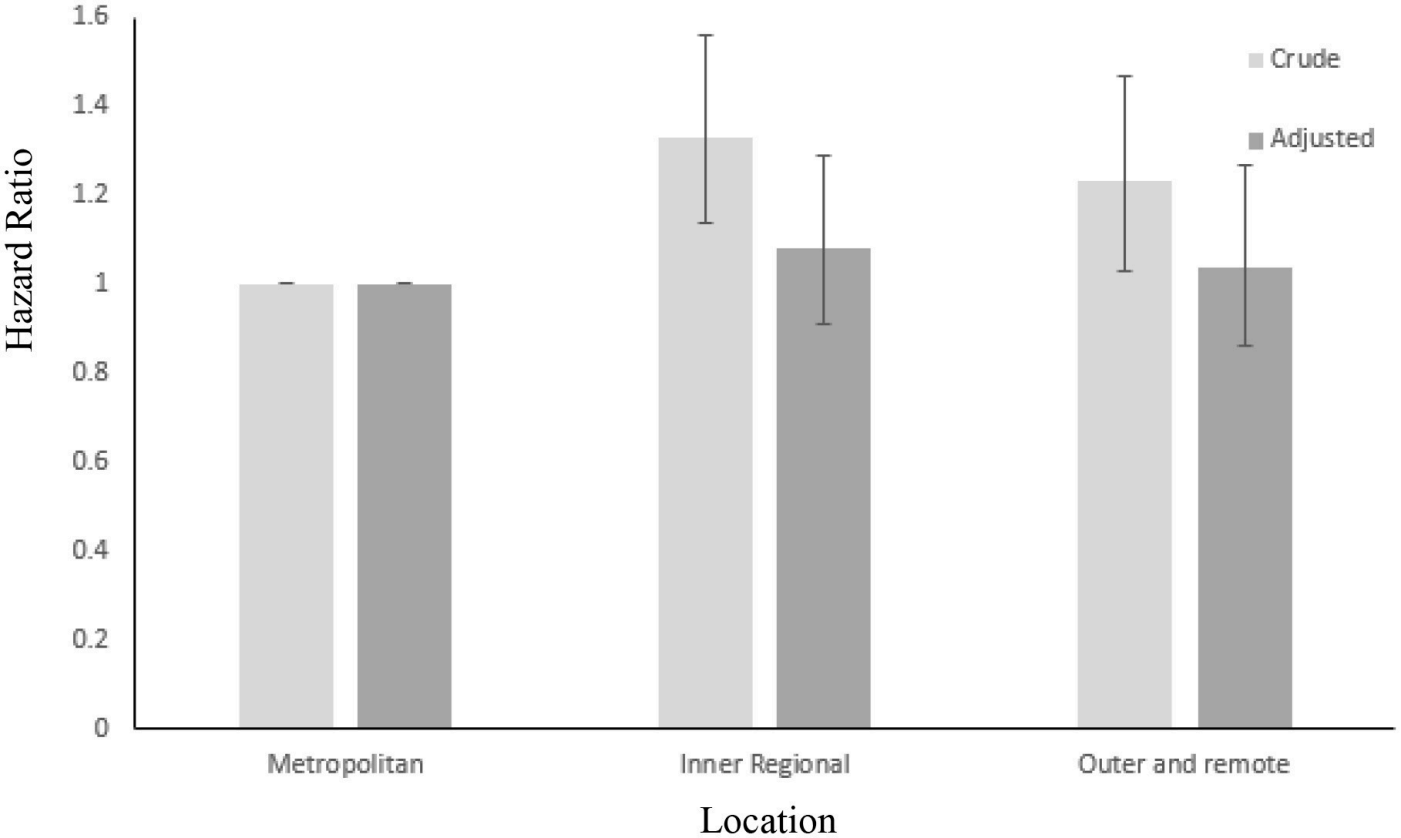
Crude and adjusted (for age and SES) hazard ratios for all – cause mortality over 10 years in metropolitan, inner regional and outer regional and remote CRC patients. Error bars: 95% CIs.

No significant differences were found between CRC specific 10 years mortality^2^ and patient place of residence. However, when controlling for stage of cancer at diagnosis, residing in a regional area was associated with a higher risk of *all-cause* mortality at 10 years post diagnosis when compared to metropolitan patients (HR = 1.33, 95% CI = 1.14–1.56). This effect was reduced slightly but remained significant when adjusted for age (HR = 1.27, 95% CI = 1.09–1.47), however as shown in Figure 2, was insignificant when adjusted for age and SES (HR = 1.08, 95% CI = 0.91–1.29). Crude comparison of metropolitan and rural patients showed higher survival in metropolitan areas (HR = 1.23, 95% CI = 1.03–1.47). This result did not change when adjusted for age, but was non-significant when adjusted for SES (HR = 1.04, 95% CI = 0.86–1.27). The same pattern of results was found when comparing rural and regional patients (HR = 0.63, 95% CI = 0.48–0.82, adjusted for age (HR = 0.67, 95% CI = 0.51–0.88) and age and SES (HR = 0.86, 95% CI = 0.63–1.14).

Diagnoses of other heart conditions (HR = 1.15, 95% CI = 0.96–1.37) and any (HR = 1.01, 95% CI = 0.82–1.23) or multiple (HR = 0.97, 95% CI = 0.66–1.42) respiratory diseases were not related to all-cause mortality at 10 years post diagnosis. Heart attack (HR = 1.164, 95% CI = 1.33–2.04) and emphysema (marginally; HR = 1.43, 95% CI = 0.99–2.07) were associated with lower 10 years survival. Heart attack diagnosis partially mediated the relationship between regional status and all-cause 10 years survival (*z* = 2.14(0.11), *p* < 0.05).

## Discussion

The current study compared previously diagnosed health conditions in metropolitan, regional, and rural CRC patients and investigated whether geographical disparity existed above and beyond the effects of age and SES. Findings suggest that place of residence is largely unrelated to the previous diagnosis of other serious health conditions, however, some disparities were apparent for certain conditions, namely, cardio-vascular and respiratory health conditions were more common in inner regional areas. The findings also indicated that CRC patients living in regional Australia experience lower 10-years survival from all causes.

The regional disparity in previously diagnosed health conditions identified here were mostly explained by older age and lower SES in inner regional locations, suggesting that the demographic profile of rural and regional areas play a key role in health disparities in non-metropolitan areas. It has long been established that low SES is associated with poorer health (20, 32). Educational, financial, and social disadvantage can often result in depleted resources for leading a healthy lifestyle and addressing health issues (33–35). Our findings support a growing body of recent evidence suggesting that regional disparity in health may be less about location and more about the characteristics of people within a geographic region (36–38). Recently, health researchers have suggested a shift in focus from broad urban-rural health disparities to understanding the unique characteristics of different communities and the individuals within them (36, 39, 40). To make the greatest improvements to the health of regional populations, it is recommended that health interventions be targeted at, and tailored toward, lower SES individuals and communities (41, 42). The current findings support this recommendation and suggest its application in CRC specific contexts. Although the association between age and mortality is unalterable, our findings raise further awareness of the aging population of our rural and regional communities and the need for focus on age appropriate health services and interventions in these areas.

In the current study, regional patients were more likely than both metropolitan and rural patients to report a previous diagnosis of certain heart conditions, one of several respiratory diseases, or multiple respiratory diseases. Research in general (not CRC-specific) adult populations has yielded similar results to those found here, highlighting that geographical disparities in health conditions are not unique to CRC patients. For example, AIHW data from a similar time period (2004–2005) shows that in general, Australians living in regional areas are significantly more likely to report various respiratory and heart problems (23). Results also suggested that higher mortality in regional CRC patients was partially attributed to their higher risk of heart attack which is not surprising considering the high risk of heart attack recurrence in any population (43). Although this finding highlights the ill-effects of regional health disparities on survival in general, it does not provide evidence of impact on CRC-*specific* survival. Nevertheless, considering the common lifestyle factors that lead to both cardiovascular and colorectal disease (i.e., diet and exercise), these findings strengthen the importance of targeting lifestyle change for improving chronic disease outcomes in regional areas (3, 5).

Despite previous evidence for an association between increasing remoteness and poor health (2, 6–10), rural CRC patients in this study (those living in the most remote areas) were no more likely to report a previously diagnosed condition, and were less likely to report previously diagnosed heart, or one or multiple respiratory conditions, than metropolitan and regional patients. This finding was unexpected considering substantial evidence suggesting rural residents report poorer health and shorter life expectancy than those living in major cities (6, 44). The better health reported by patients in rural communities was for the most part, *not* accounted for by differences in age or SES and therefore may potentially be due to environmental factors unique to rural living that could be investigated in future research. Alternatively, the prevalence of other chronic health conditions in remote areas may appear lower, as residents living further away from health services are less likely to access medical attention (45, 46) and therefore less likely to have certain health conditions identified and diagnosed. Sampling error is another potential explanation for unexpected findings and replication in a different sample may be warranted before solid conclusions can be drawn.

### Limitations

The current study was conducted using a large representative sample of CRC patients, however, some limitations exist. For example, individual data on SES was not available, meaning the estimates were based on the status of each local government area as a whole. This allowed us to draw inferences based on the average SES of a community but did not allow us to study individual SES; a method that would result in more precise estimates. Similarly, information on participant ethnic identification was not collected and this prevented us from assessing its role. Finally, our sample of outer regional and remote participants was small in comparison to other groups. Although this is representative of the Australian population, it likely explained their higher variance in outcomes. Equal group sizes may have led to the emergence of more significant effects and future research might benefit from over-representation of participants in more remote locations. A final consideration to note when interpreting findings is that the current sample is particularly reflective of younger, earlier stage colon cancer patients.

### Conclusion

Our findings suggest that very few geographical disparities in previously diagnosed health conditions are apparent among CRC patients and those that do exist are largely explained by age and SES, rather than geographical location itself. In addition, lower CRC-specific survival rates in inner regional areas do not appear to be impacted by the limited disparities in previously diagnosed health conditions that do exist. Public health initiatives and health services aiming to increase the overall health of CRC patients and reduce mortality, should be targeted at lower SES and aging communities and will benefit from addressing lifestyle changes that impact more than one chronic health condition.

## Ethics statement

The study used archival data gathered as part of the Colorectal Cancer and Quality of Life study, which was approved by the University of Queensland's Behavioral and Social Science Ethical Review Committee. Participants provided informed consent via mail. The University of Southern Queensland's Human Research Ethics Committee approved the use of this data (H17-REA014).

## Author contributions

BG formulated the research question, performed the bulk of data analysis, and drafted the full manuscript. SM provided substantial input in planning and revising the manuscript. MI provided statistical support and contributed substantial revision to the results section of the manuscript. FC-W contributed content and revisions to the introduction and the discussion. SN conducted cluster analyses and authored parts of the methods section. PB was heavily involved in data collection and management. SC was instrumental in the research design and data collection phases of the research and contributed substantial revision to the final manuscript. JA played a key role in the design of the research, co-ordination of data collection and formulating the research question. JD also played a key role in the design of the research and provided substantial revision to the manuscript.

### Conflict of interest statement

The authors declare that the research was conducted in the absence of any commercial or financial relationships that could be construed as a potential conflict of interest.

## Footnotes

^1^With the exception of “*heart attack”, “stroke”*, and “*angina pectoris”*, items reflected pervasive, chronic conditions that, although likely to be comorbid with the colorectal diagnosis, cannot be assumed as such hence we refer to as previously diagnosed conditions, rather than comorbid conditions.

^2^5-year mortality was also measured and no significant differences were found for patient place of residence.

## References

[1] Begg SJ Vos T Barker B Stanley L Lopez AD. Burden of disease and injury in Australia in the new millennium: measuring health loss from diseases, injuries and risk factors. Med J Aust. (2008) 188:36. Available Online at: www.mja.com.au/system/files/issues/188_01_070108/beg10596_fm.pdf 18205562 10.5694/j.1326-5377.2008.tb01503.x

[2] Cramb S Mengersen K Baade PD Atlas of Cancer in Queensland: Geographical Variations in Incidence and Survival 1998-2007: Viertel Centre for Research in Cancer Control. Cancer Coucil Queensland (2011).

[3] Crouch R Wilson A Newbury J. A systematic review of the effectiveness of primary health education or intervention programs in improving rural women's knowledge of heart disease risk factors and changing lifestyle behaviours. Int J Evid Based Healthcare (2011) 9:236–45. 10.1111/j.1744-1609.2011.00226.x 21884451

[4] Ireland MJ March S Crawford-Williams F Cassimatis M Aitken JF Hyde MK. A systematic review of geographical differences in management and outcomes for colorectal cancer in Australia. BMC Cancer (2017) 17:95. 10.1186/s12885-017-3067-1 28152983 PMC5290650

[5] Eberhardt MS Pamuk ER. The importance of place of residence: Examining health in rural and nonrural areas. Am J Public Health (2004) 94:1682–6. 10.2105/AJPH.94.10.1682 15451731 PMC1448515

[6] Smith KB Humphreys JS Wilson MG. Addressing the health disadvantage of rural populations: how does epidemiological evidence inform rural health policies and research? Aust J Rural Health (2008) 16:56–66. 10.1111/j.1440-1584.2008.00953.x 18318846

[7] Australian Institute of Health and Welfare Cancer in Australia: An overview 2006. Australian Institute of Health and Welfare (2007).

[8] Schouten L Meijer H Huveneers J Kiemeney L. Urban-rural differences in cancer incidence in the Netherlands, 1989–1991. Int J Epidemiol. (1996) 25:729–36. 10.1093/ije/25.4.729 8921449

[9] O'Connor A Wellenius G. Rural–urban disparities in the prevalence of diabetes and coronary heart disease. Public Health (2012) 126:813–20. 10.1016/j.puhe.2012.05.029 22922043

[10] Pong RW DesMeules M Lagacé C. Rural–urban disparities in health: How does Canada fare and how does Canada compare with Australia? Aust J Rural Health (2009) 17:58–64. 10.1111/j.1440-1584.2008.01039.x 19161503

[11] Booth H Mortality Atlas, Australia: 1997 to 2000. JSTOR (2003).

[12] Phillips A. Health status differentials across rural and remote Australia. Aust J Rural Health (2009) 17:2–9. 10.1111/j.1440-1584.2008.01029.x 19161493

[13] Jong KE Smith DP Yu XQ O'Connell DL Goldstein D Armstrong BK. Remoteness of residence and survival from cancer in New South Wales. Med J Aust. (2004) 180:618–22. Available Online at: www.mja.com.au/system/files/issues/180_12_210604/jon10431_fm.pdf 15200358 10.5694/j.1326-5377.2004.tb06123.x

[14] Wilkinson D Cameron K. Cancer and cancer risk in South Australia: what evidence for a rural–urban health differential? Aust J Rural Health (2004) 12:61–6. 10.1111/j.1038-5282.2004.00555.x 15023223

[15] Dixon J Welch N. Researching the rural–metropolitan health differential using the ‘social determinants of health'. Aust J Rural Health (2000) 8:254–60. 10.1111/j.1440-1584.2000.tb00366.x 11894255

[16] Beckmann KR Bennett A Young GP Cole SR Joshi R Adams J. Sociodemographic disparities in survival from colorectal cancer in South Australia: a population-wide data linkage study. BMC Health Services Res. (2016) 16:24. 10.1186/s12913-016-1263-3 26792195 PMC4721049

[17] Chawla N Butler EN Lund J Warren JL Harlan LC Yabroff KR. Patterns of colorectal cancer care in Europe, Australia, and New Zealand. J Natl Cancer Inst Monographs (2013) 2013:36–61. 10.1093/jncimonographs/lgt009 23962509 PMC3888187

[18] Edwards BK Noone AM Mariotto AB Simard EP Boscoe FP Henley SJ. Annual Report to the Nation on the status of cancer, 1975-2010, featuring prevalence of comorbidity and impact on survival among persons with lung, colorectal, breast, or prostate cancer. Cancer (2014) 120:1290–314. 10.1002/cncr.28509 24343171 PMC3999205

[19] Larson A. Rural health's demographic destiny. Rural Remote Health (2006) 6:1–8. Available Online at: https://www.rrh.org.au/journal/article/551 16623618

[20] Adler NE Boyce T Chesney MA Cohen S Folkman S Kahn RL. Socioeconomic status and health: The challenge of the gradient. Am Psychol. (1994) 49:15. 10.1037/0003-066X.49.1.15 8122813

[21] Baade PD Dasgupta P Aitken JF Turrell G. Geographic remoteness, area-level socioeconomic disadvantage and inequalities in colorectal cancer survival in Queensland: a multilevel analysis. BMC Cancer (2013) 13:493. 10.1186/1471-2407-13-493 24152961 PMC3871027

[22] Lynch BM Baade P Fritschi L Leggett B Owen N Pakenham K.. Modes of presentation and pathways to diagnosis of colorectal cancer in Queensland. Med J Aust. (2007) 186:288–91. Available Online at: https://www.mja.com.au/system/files/issues/186_06_190307/lyn11021_fm.pdf 17371208 10.5694/j.1326-5377.2007.tb00902.x

[23] Australian Insitute of Health and Welfare Rural, Regional and Remote Health: Indicators of Health Status and Determinants of Health. Canberra, ACT: Australian Institute of Health and Welfare Canberra (2008).

[24] Ng SK Holden L Sun J. Identifying comorbidity patterns of health conditions via cluster analysis of pairwise concordance statistics. Statist Med. (2012) 31:3393–405. 10.1002/sim.5426 22714868

[25] Prados-Torres A Calderón-Larranaga A Hancco-Saavedra J Poblador-Plou B van den Akker M. Multimorbidity patterns: a systematic review. J Clin Epidemiolo. (2014) 67:254–66. 10.1016/j.jclinepi.2013.09.021 24472295

[26] Batstra L Bos E Neeleman J. Quantifying psychiatric comorbidity Lessions from chronic disease epidemiology. Soc Psychiatry Psychiatr Epidemiol. (2002) 37:105–11. 10.1007/s001270200001 11995637

[27] Baty F Putora PM Isenring B Blum T Brutsche M. Comorbidities and burden of COPD: a population based case-control study. PloS ONE (2013) 8:e63285. 10.1371/journal.pone.0063285 23691009 PMC3656944

[28] Benjamini Y Hochberg Y Controlling the false discovery rate: a practical and powerful approach to multiple testing. J R Statis Soc Series B. (1995):289–300.

[29] Borgatti SP Everett MG Freeman LC Ucinet forWindows: Software for Social Network Analysis. Harvard, MA: Analytic Technologie (2002).

[30] Corp I. IBM SPSS Statistics for Windows, Version 23.0. Armouk, NY: IBM Corp (2014).

[31] MacKinnon DP Krull JL Lockwood CM. Equivalence of the mediation, confounding and suppression effect. Prevent Sci. (2000) 1:173–81. 10.1023/A:1026595011371 11523746 PMC2819361

[32] Meyer OL Castro-Schilo L Aguilar-Gaxiola S. Determinants of mental health and self-rated health: a model of socioeconomic status, neighborhood safety, and physical activity. Am J Public Health (2014) 104:1734–41. 10.2105/AJPH.2014.302003 25033151 PMC4151927

[33] Pickett KE Wilkinson RG. Income inequality and health: a causal review. Soc Sci Med. (2015) 128:316–26. 10.1016/j.socscimed.2014.12.031 25577953

[34] Veenstra G. Social capital, SES and health: an individual-level analysis. Soc Sci Med. (2000) 50:619–29. 10.1016/S0277-9536(99)00307-X 10658843

[35] Winkleby MA Jatulis DE Frank E Fortmann SP. Socioeconomic status and health: how education, income, and occupation contribute to risk factors for cardiovascular disease. Am J Public Health (1992) 82:816–20. 1585961 10.2105/ajph.82.6.816 PMC1694190

[36] Hartley D. Rural health disparities, population health, and rural culture. Am J Public Health (2004) 94:1675–8. 10.2105/AJPH.94.10.1675 15451729 PMC1448513

[37] Heathcote KE Armstrong BK editors Disparities in Cancer Outcomes in Regional and Rural Australia. Cancer Forum: The Cancer Council Australia (2007).

[38] Sun J March S Ireland MJ Crawford-Williams F Goodwin BC Hyde MK. Socio-demographic factors drive regional differences in participation in the National Bowel Cancer Screening Program (NBCSP) – An ecological analysis on the NBCSP data. Aust N Z J Public Health (2017) 42:92–7. 10.1111/1753-6405.12722 29044846

[39] Cohen SA Cook SK Kelley L Foutz JD Sando TA. A closer look at rural-urban health disparities: associations between obesity and rurality vary by geospatial and sociodemographic factors. J. Rural Health (2017) 33:167–79. 10.1111/jrh.12207 27557442

[40] Eley R Bush R Brown W. Opportunities, barriers, and constraints to physical activity in rural Queensland, Australia. J Phys Act Health (2014) 11:68–75. 10.1123/jpah.2011-0312 23250028

[41] Bukman AJ Teuscher D Feskens EJ van Baak MA Meershoek A Renes RJ. Perceptions on healthy eating, physical activity and lifestyle advice: opportunities for adapting lifestyle interventions to individuals with low socioeconomic status. BMC Public Health (2014) 14:1036. 10.1186/1471-2458-14-1036 25280579 PMC4210550

[42] Teuscher D Bukman AJ Meershoek A Renes RJ Feskens EJ van Baak MA. Adapting an effective lifestyle intervention towards individuals with low socioeconomic status of different ethnic origins: the design of the MetSLIM study. BMC Public Health (2015) 15:125. 10.1186/s12889-015-1343-z 25880746 PMC4339423

[43] Fuster V Walsh Harrington R Hurst's the Heart. 13th ed New York, NY: McGraw Hill Professional (2011).

[44] Singh GK Siahpush M. Widening rural–urban disparities in life expectancy, US, 1969–2009. Am J Prevent Med. (2014) 46:e19–29. 10.1016/j.amepre.2013.10.017 24439358

[45] Ansari Z Laditka JN Laditka SB. Access to health care and hospitalization for ambulatory care sensitive conditions. Med Care Res Rev. (2006) 63:719–41. 10.1177/1077558706293637 17099123

[46] Jordan H Roderick P Martin D Barnett S. Distance, rurality and the need for care: access to health services in South West England. Int J Health Geograph. (2004) 3:21. 10.1186/1476-072X-3-21 15456514 PMC524184

